# Spiral-Shaped Piezoelectric MEMS Cantilever Array for Fully Implantable Hearing Systems

**DOI:** 10.3390/mi8100311

**Published:** 2017-10-18

**Authors:** Péter Udvardi, János Radó, András Straszner, János Ferencz, Zoltán Hajnal, Saeedeh Soleimani, Michael Schneider, Ulrich Schmid, Péter Révész, János Volk

**Affiliations:** 1 Institute for Technical Physics and Materials Science, MTA EK, 1121 Konkoly Thege M. út 29-33, H-1121 Budapest, Hungary; udvardi.peter98@gmail.com (P.U.); rado@mfa.kfki.hu (J.R.); straszner@mfa.kfki.hu (A.S.); ferencz@mfa.kfki.hu (J.F.); hajnal@mfa.kfki.hu (Z.H.); soleimani.saeedeh@energia.mta.hu (S.S.); 2 Doctoral School on Material Sciences and Technologies, Óbuda University, Bécsi út 96/b, H-1034 Budapest, Hungary; 3 Institute of Sensor and Actuator Systems, TU Wien, 1040 Vienna, Austria; michael.schneider@tuwien.ac.at (M.S.); ulrich.e366.schmid@tuwien.ac.at (U.S.); 4 Department of Otorhinolaryngology-Head and Neck Surgery, Clinical Center, University of Pécs, H-7601 Pécs, Hungary; revesz.peter@pte.hu

**Keywords:** artificial basilar membrane, cochlear implant, frequency selectivity, Archimedean spiral, aluminum nitride (AlN), piezoelectric cantilever, micro-electromechanical system (MEMS), finite element analysis, energy harvesting

## Abstract

Fully implantable, self-powered hearing aids with no external unit could significantly increase the life quality of patients suffering severe hearing loss. This highly demanding concept, however, requires a strongly miniaturized device which is fully implantable in the middle/inner ear and includes the following components: frequency selective microphone or accelerometer, energy harvesting device, speech processor, and cochlear multielectrode. Here we demonstrate a low volume, piezoelectric micro-electromechanical system (MEMS) cantilever array which is sensitive, even in the lower part of the voice frequency range (300–700 Hz). The test array consisting of 16 cantilevers has been fabricated by standard bulk micromachining using a Si-on-Insulator (SOI) wafer and aluminum nitride (AlN) as a complementary metal-oxide-semiconductor (CMOS) and biocompatible piezoelectric material. The low frequency and low device footprint are ensured by Archimedean spiral geometry and Si seismic mass. Experimentally detected resonance frequencies were validated by an analytical model. The generated open circuit voltage (3–10 mV) is sufficient for the direct analog conversion of the signals for cochlear multielectrode implants.

## 1. Introduction

Cochlear implant (CI) is a surgically implanted electronic device that provides a sense of sound to a person who suffers from profound hearing loss or deafness. The present generation of hearing systems bypass the normal hearing process. Outside the skin, it consists of a microphone, a speech processor, and a transmitter, which transmits signals through an internal receiver to an array of electrodes placed in the cochlea. Though, in the last three decades the technology has undergone an impressive improvement, there are still some challenges to be addressed for higher wearing comfort [1]. Because of the external units, the system is visible, making the patients stigmatized. It also limits the activities that can be undertaken while wearing the device. Broken wires, cables, and speech processors can cause derangement, too. This could be minimized by having a fully implantable cochlear implant (FICI, sometimes also referred to as totally implantable cochlear implant, TICI) which functions round-the-clock while sleeping, showering, swimming, and during many types of vigorous physical activities [2]. FICI is supposed to be composed of an internal microphone or a piezoelectric acoustic sensor, an electronic device which transform the signal for the hearing nerves, a battery and/or an energy harvesting unit, as well as a multielectrode array inserted in the cochlea.

Several solutions have been proposed to mimic the frequency selectivity (tonotopy) of the cochlea. The topologically more faithful physical model is an elastic membrane having varying widths along its length [3,4,5,6,7]. The other approach is to apply an array of micro-electromechanical system (MEMS) cantilevers having varying length, and thus, varying natural resonance frequency. Because of the smaller size and more reliable fabrication procedure, the latter approach seems to have higher potential for FICI. In 1998 Harada et al. demonstrated a fishbone structured acoustic sensor using piezoresistive read-out elements [8]. Later, Xu et al. reported polymeric micro-cantilever array to mimic the mammalian cochlea [9]. Recently, Jang et al. used an aluminum nitride (AlN) coated array as an artificial basilar membrane [10,11]. Though the rectangular unimorph cantilevers showed excellent frequency selectivity, they covered only the upper half of the human hearing range (2.9–12.6 kHz) [11], since the natural resonance frequency at fixed cantilever thickness scales up with decreasing length. To obtain sensitive cantilevers in the range of 300–700 Hz is, however, more challenging.

Low frequency, spiral, and spiral-like cantilevers were proposed and theoretically evaluated by Choi et al. in 2006 [12], and recently, also experimentally demonstrated. Zhang et al. [13], and Lu et al. [14] reported an S-shaped Lead Zirconate Titanate (PZT) coated flexure suspended MEMS device on a chip size of 6 mm × 6 mm, for vibration sensing and energy harvesting. Though the covered frequency range is attractive for the proposed FICI concept, a smaller footprint, higher Q-factor (>100), and bio- and metal-oxide-semiconductor (CMOS) compatible piezoelectric material, like AlN [15,16], are needed for the device to be implantable in the human middle ear.

Voice detection in CIs is done directly by perceiving the modulation of air or fluid pressure using a microphone, even if the functions of the tympanic membrane and of the middle ear are intact. An alternative approach, also an aim in this paper, is to measure the vibration of the ossicles with a miniaturized MEMS based implant. As a guideline for the design, we referred to the work of Gan et al. [17] on implantable middle ear hearing devices (IMEHDs). In their work, a small magnet was mounted between the malleus and stapes, and driven electromagnetically by a coil placed under the ear canal bony wall. The diameter and length of the cylinder-shaped magnet is 1.5 and 2 mm, respectively, and it weighs 26 mg, which is comparable to the size and mass of a 3D packaged Si multicantilever system. Beker et al. demonstrated a rectangular cantilever with Si seismic mass to achieve resonance in this lower voice frequency range (474 Hz) [18]. However, the size (6 mm × 6 mm) and weight of the chip, especially in packaged multichannel form, is too large to be fixed onto one of the middle ear bones. Moreover, the applied technique to bond bulk piezoceramic PZT dices to the substrate, and the grinding, make the wafer scale processing highly demanding.

Here, we demonstrate an array of spiral cantilevers with Si seismic mass at their ends, which is optimized to achieve a small footprint, compactness, bio- and CMOS compatibility, low resonance frequency (300–700 Hz), high Q-factor in air (117–254), and high robustness with limited internal stress in the Si cantilever. These 2 mm × 2 mm cantilever chips are small enough to pack them in a compact multichannel device which fits into the middle auditory system, and can provide a new solution for next generation FICI systems.

## 2. Materials and Methods 

### 2.1. Cantilever Design 

According to the Euler–Bernoulli theory, the first natural frequency (*n* = 1) of a one-side-clamped rectangular Si cantilever is inversely proportional to the square of the length (*L*) of the beam [19]:

(1) $$f_{\mathrm{beam}}=\frac{\beta_{1}^{2}}{2\pi }\sqrt{\frac{E_{\mathrm{Si}}}{12\rho_{\mathrm{Si}}}}\frac{t}{L^{2}},$$

where *β*_1_ = 1.875, *t* is thickness of the cantilever, *E*_Si_ is the Young modulus for <110> crystallographic orientation (1.69 × 10^11^ Pa) [20] and *ρ*_Si_ is the mass density (2330 kg/m^3^) of Si. It means that using a Si-on-Insulator (SOI) wafer, with a typical Si device thickness of 12.5 μm and a cantilever length of 7.6 mm, is needed to reach the lower end of the voice frequency range (300 Hz). This size is too large to allow implantation of the device in the inner ear. However, by applying a tip mass (*M*) at the free end of the beam with a distributed mass (*m*), the first natural frequency can be reduced as follows [21]:

(2) $$f_{\mathrm{tm}}=\frac{1}{2\pi }\sqrt{\frac{3E_{\mathrm{Si}}Wt^{3}}{12\left(M+0.24m\right)L^{3}}}.$$

If we assume that the tip mass is significantly higher than that of the cantilever (*M* ≫ *m*) the reduced frequency due to the tip mass can be approximated by

(3) $$f_{\mathrm{tm}}=\sqrt{\frac{m}{M}}f_{\mathrm{beam}}.$$

Moreover, the seismic mass also helps to minimize the air damping effect, and increases the stored energy [12]. However, too large a mass results in a high internal stress, and a fracture of the cantilever upon resonance. Therefore, a trade-off is needed between cantilever length and the proof mass.

The one side clamped spiral cantilevers were designed to fit into a 2 mm *×* 2 mm square window. The thickness of the single crystal Si beam is 12.5 μm, which corresponds to the device layer of the selected SOI wafer. The arc of the beam was defined by the parametric equation of an Archimedean spiral, where the curvature radius is continuously decreasing, with the azimuth angle φ from an initial radius at the clamping point (*R*_0_), until reaching the radius of the proof mass (*r*_0_),

(4) $$\left(\begin{array}{c} x\\ y \end{array}\right)=\left(R_{0}-c\varphi \right)\left(\begin{array}{c} \cos\left(\varphi \right)\\ \sin\left(\varphi \right) \end{array}\right).$$

**Figure 1:**
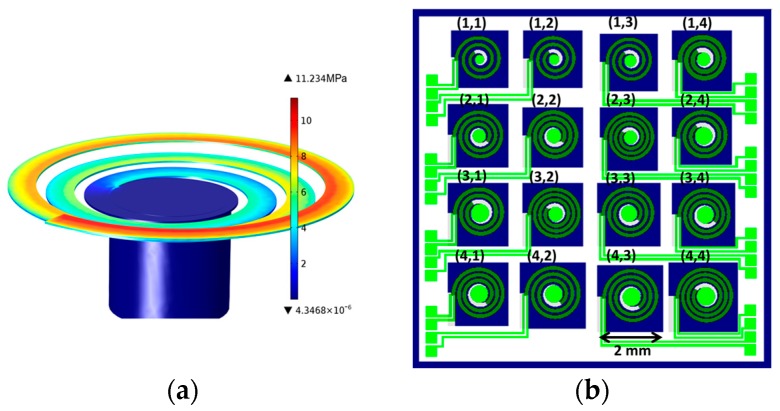
(**a**) Tensile stress distribution along the beam calculated by finite element analysis to exclude “fragile” geometries upon selection; (**b**) Layout of the selected 4 × 4 spirals. Each of the spirals fit into a square area of 2 mm × 2 mm. Five geometrical parameters of the Archimedean spirals were varied to tune the natural frequency: number of the half turns, width (W), starting radius at the clamping point (*R*_0_), radius of the proof mass cylinder (*r*_0_), and *c* parameter, describing the rate of the radius change from the edge towards the center.

Finite element analysis, using COMSOL Multiphysics (5.2a, Burlington, MA, USA ), was used to select 16 different geometries which fulfilled the following two requirements: (i) the (first) significant natural resonance has to fall in the frequency range of 300–1000 Hz; and (ii) the maximal von Mises stress along the cantilever at a driving acceleration of 1 g is not allowed to exceed 5% of the fracture strength of anisotropically etched Si diaphragms, i.e., 300 MPa [22]. One typical stress distribution in Figure 1a shows that the stress is gradually decreasing from the clamping side toward the tip mass without reaching the chosen limit of 15 MPa. Several spiral geometries were tested numerically by changing the parameters as follows: width of the cantilever in the range of 140–180 μm, the number of the turns between 3 and 4 φ = 6–8π), c parameter in a range of 27.0–36.5 μm/rad, and the radius of the tip mass between 160 to 300 μm. The selected 16 spirals are shown in Figure 1b.

Since the polarization axis of AlN thin films (*c*-axis) is nearly perpendicular to the substrate, a *d*_31_ type contacting scheme was used (top-bottom) to generate electrical signals. The piezo layer does not fully cover the whole length of the arc, as the last segment towards the seismic mass is almost stress-free.

### 2.2. Analytical Model 

The model was based on the one described by Karami et al. [23], with a few modifications to include the effects of the tip mass attached to the spiral cantilever. Therefore, the kinetic energy *T* and the potential energy *V* for the system are

(5) $$T=\int \frac{1}{2}\rho A{\left(\frac{\partial w}{\partial t}\right)}^{2}+\frac{1}{2}I_{z}{\left(\frac{\partial \beta }{\partial t}\right)}^{2}dx+\frac{1}{2}M{\left(\frac{\partial w}{\partial t}\right)}^{2}{|}_{x=L}+\frac{1}{2}I_{t_{x}}{\left(\frac{\partial^{2}w}{\partial x\partial t}\right)}^{2}{|}_{x=L}\;+\frac{1}{2}I_{t_{z}}{\left(\frac{\partial \beta }{\partial t}\right)}^{2}{|}_{x=L}$$

(6) $$V=\int \frac{1}{2}\left[EI{\left(\frac{\beta }{R}-\frac{\partial^{2}w}{\partial x^{2}}\right)}^{2}+GJ{\left(\frac{\partial \beta }{\partial x}+\frac{1}{R}\frac{\partial w}{\partial x}\right)}^{2}\right]dx$$

where *w*(*x,t*) is the deflection, *β*(*x,t*) is the twist angle of the cantilever, *R*(*x*) is the radius of the spiral, *A* is the area of the cantilever, *E* is the composite Young modulus, *I* is the second moment of inertia, *J* is the torsion constant, *G* is the shear modulus (modulus of rigidity), *I_tx_* and *I_tz_* are the mass moment of inertia of the seismic mass with respect to *x* and *z* axis. To account for the AlN (*E*_AlN_ = 344 GPa) and Au (*E*_AlN_ = 79 GPa) [24] layers deposited on top of the Si, the Young’s modulus in the calculations was taken for the composite material as described in Timoshenko and Young’s textbook [25,26]. For the thicker (12.5 μm) Si membrane, the crystal orientation dependence of Si can also play a role. Since the in-plane orientation of the spiral alternate between <100> and <110> directions along the spiral arc with an angular periodicity of π/2, the average of the corresponding Young moduli (130 GPa and 169 GPa, respectively [26]) was taken into account in the model. Thus, the obtained composite Young modulus for Equation (6) was *E* = 212 GPa.

Equating the variation of the action (∫T−Vdt) to be zero yields six equations, from which only four constrain the system on the spatial boundaries, and the solutions are only functions of time. Hence, transforming all six equations into frequency domains yields two coupled equations for the spatial coordinates, which are as follows,

(7) $$\omega^{2}\rho Aw+\frac{d^{2}}{dx^{2}}EI\left[\frac{\beta }{R}+\frac{d^{2}w}{dx^{2}}\right]+\frac{d}{dx}\frac{GJ}{R}\left[\frac{d\beta }{dx}+\frac{1}{R}\frac{dw}{dx}\right]=\omega^{2}\rho Aw_{b},$$

(8) $$-\omega^{2}I_{z}\beta +\frac{EI}{R}\left[\frac{\beta }{R}-\frac{d^{2}w}{dx^{2}}\right]-\frac{d}{dx}GJ\left[\frac{d\beta }{dx}+\frac{1}{R}\frac{\partial w}{dx}\right]=0,$$

as well as four equations, which provide natural boundary conditions for the equations above:

(9) $${\left[-\omega^{2}Mw+\frac{d}{dx}EI\left[\frac{\beta }{R}-\frac{d^{2}w}{dx^{2}}\right]+\frac{GJ}{R}\left[\frac{d\beta }{dx}+\frac{1}{R}\frac{dw}{dx}\right]\right]}_{x=L}=0$$

(10) $${\left[EI\left[\frac{\beta }{R}-\frac{d^{2}w}{dx^{2}}\right]+\omega^{2}I_{t_{x}}\frac{dw}{dx}\right]}_{x=L}=0$$

(11) $${\left[-\omega^{2}I_{t_{z}}\beta +GJ\left[\frac{d\beta }{dx}+\frac{1}{R}\frac{dw}{dx}\right]\right]}_{x=L}=0$$

(12) $$w\left(0\right)=\frac{dw}{dx}{|}_{x=0}=\beta \left(0\right)=0$$

The equations were solved using the Chebyshev spectral collocation method, implemented by the library of the open-source software, Chebfun. The library was chosen as it provides a quick solution, even for a high-resolution frequency sweep.

### 2.3. Fabrication

**Figure 2:**
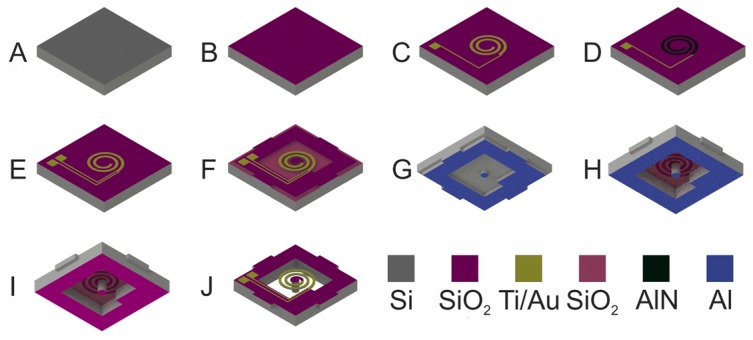
Schematics of the fabrication procedure: (**A**) 4” Si-on-Insulator (SOI) wafer; (**B**) thermal oxidation (300 nm); (**C**) deposition and lift-off of the bottom Ti/Au contact and pads; (**D**) radio frequency (RF) sputter deposition and patterned etching of aluminum nitride (AlN) (830 nm); (**E**) deposition and lift-off of top Ti/Au contact; (**F**) deep reactive ion etching (DRIE) of the spiral beam from the front side; (**G**) first DRIE Bosch process from the back-side and the strip of the photoresist mask; (**H**) second DRIE Bosch process from the back-side through the Al hard mask with a slightly modified pattern to obtain perforated Si frame; (**I**) etching of the Al masking layer; (**J**) hydrofluoric acid (HF) etching of the buried oxide and removal of the top protective photoresist layer.

The unimorph, *d*_31_ type piezocantilever arrays were fabricated on a 4” SOI wafer with 12.5 μm Si device layer, 1 μm buried oxide (BOX), and a handle layer of 550 μm. The fabrication process is shown in Figure 2. At first, thermal oxide layer with a thickness of 300 nm was grown onto the wafer (Figure 2B). The bottom electrode of Ti (30 nm)/Au (120 nm) was prepared by e-beam evaporation (AJA) and a subsequent lift-off step (Figure 2C). The piezoelectric AlN layer, having a thickness of 830 nm, was deposited by reactive radio frequency (RF) sputtering from an 8“ Al target in a Leibold Heraeus Z550 system (CAE Inc., Montreal, QC, Canada). No additional substrate heating was applied beyond the natural effect of the RF generated nitrogen plasma (500 W). The maximum substrate temperature during the deposition is around 300 °C. The AlN layer was then patterned by photolithography and wet chemical etching using phosphoric acid (Figure 2D). It was followed by the deposition of a Ti (30 nm)/Au (120 nm) top electrode at the same conditions used for its lower counter electrode (Figure 2E). The micromachining of the spiral cantilevers was started from the front side using a deep reactive ion etching (DRIE) step in an Oxford Plasmalab System 100 ICP180 (Oxford Instruments Plasma Technologies, Yatton, Bristol, UK). The etching was performed through the whole device layer of 12.5 μm, and was stopped by the BOX layer (Figure 2F). Etching from the back side was carried out by Bosch process, in such a way to obtain full wafer thick (550 μm) seismic masses at the free end of the cantilevers. In addition, it was split into two steps using, at first, photoresist, and then Al mask, with slightly different patterns to obtain perforated Si wafers, in which the 4 × 4 block arrays are connected by thinned (~150 μm) Si bridges (Figure 2G,H). The aim of this method was to avoid chip dicing, which would have damaged the sensitive cantilevers. The wafer process was followed by etching of the Al hard mask in phosphoric acid (Figure 2I), and finished by the wet etching of the buried oxide in hydrofluoric acid, to release the spiral cantilevers (Figure 2J). The manually cleaved dices were firmly mounted, and wire bonded onto a printed circuit board (PCB) recessed under the cantilever, which ensured the electrical readout and the free vibration of the piezo cantilevers.

### 2.4. Characterization

**Figure 3:**
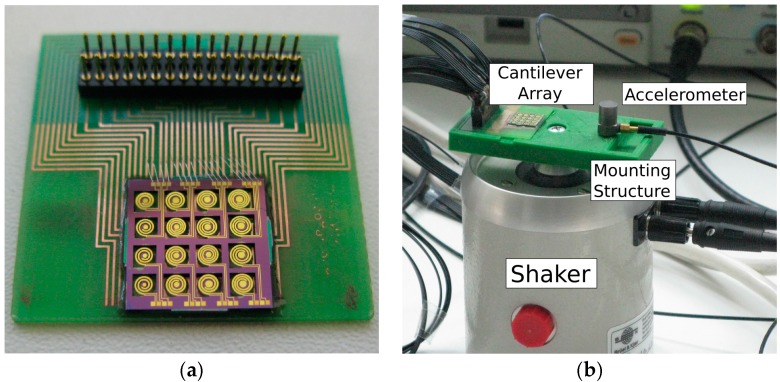

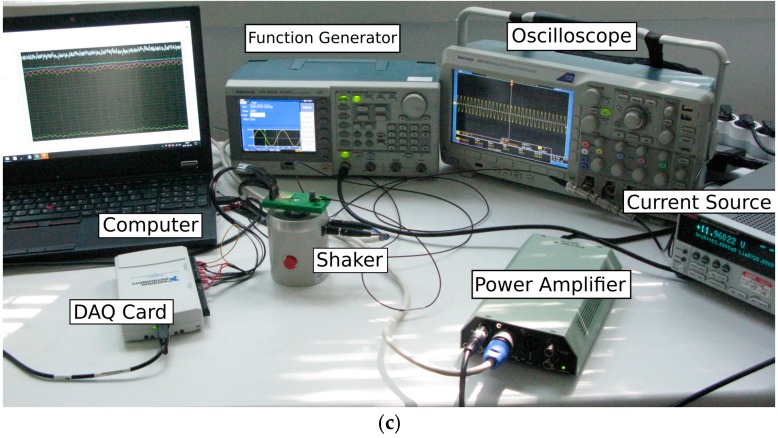
Experimental setup for the characterization of the piezoelectric cantilever arrays: (**a**) Wire bonded cantilever array on the printed circuit board (PCB); (**b**) 3D printed sample holder with a calibrated accelerometer mounted on a shaker; (**c**) the shaker was controlled by a function generator through a power amplifier. The output voltage signals of the cantilevers and the calibrated accelerometer were collected by a programmed data acquisition card (DAQ). The signals were in situ visualized by an oscilloscope during the automatized frequency sweeps. Current source was used to feed the accelerometer.

The fabricated piezoelectric cantilevers were analyzed in a LEO XB-1540 crossbeam scanning electron microscope (SEM) (Zeiss, Oberkochen, Germany) and in a New View 7100 optical surface profiler (ZYGO, Middlefield, CT, USA). During the electromechanical tests, the PCB (Figure 3a) was placed on a purpose designed 3D printed chip holder mounted with a miniaturized calibrated accelerometer (4397-A, Brüel & Kjaer, Nærum, Denmark) (Figure 3b). Vibrations were carried out by a mini shaker (LDS V201) which was driven by a power amplifier (2735, Brüel & Kjaer) controlled by signal generator (AFG 3252C, Tectronix, Beaverton, OR, USA) (Figure 3c). LabView software was written to perform frequency sweeps and collect open circuit voltage (V_OC_) signals through a data acquisition card (USB DAQ 6211, National Instruments, Austin, TX, USA). The amplitude of the generated sinusoidal signal was controlled by a closed feedback loop mechanism using the signal or the accelerometer. In this way, the acceleration is fixed to a constant level during the frequency sweep. The output voltage signals were collected for each cantilever at continuous sweep of the sinusoidal excitation in the frequency range of 20 Hz–1.2 kHz. It is worth mentioning that in contrast to several studies, we did not measure the direct effect of sound pressure wave on the sensor. Instead, we applied an external acceleration on the frame of the Si chip.

## 3. Results and Discussion

### 3.1. Structural Characterization

**Figure 4:**
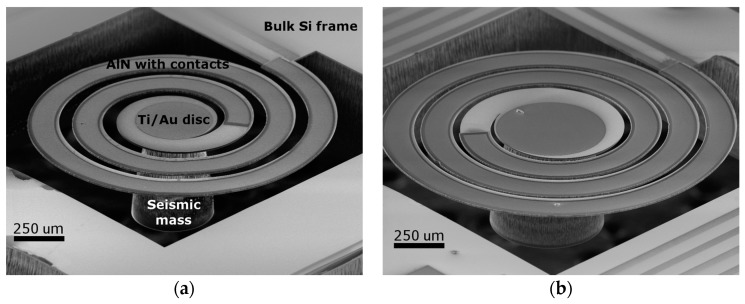
Tilt-view scanning electron microscope (SEM) images of two typical suspended spiral-shaped cantilevers (1,3) (**a**) and (3,4) (**b**) with contacted AlN layer (darker region) on their top surfaces, and a wafer thick 3D-micromachined Si seismic mass beneath. The darker circle in the center corresponds to a Ti/Au disc applied for laser reflection tests. Truncated shape of the tip mass is due to the increasing underetching ratio during the DRIE.

Most of the chips on the 4” wafer with the sensitive spirals survived the over 30-step fabrication process; an example is shown in Figure 3a. It was especially critical when releasing the cantilevers from the buried SiO_2_ membrane (Figure 2J) and during the manual dicing of the cantilever arrays. The cantilevers also tolerated the vibration test up to an instrumental acceleration limit of 5 g. Scanning electron micrograph of two typical spiral cantilevers situated in the (1,3) and (3,4) array positions are shown in Figure 4a,b, respectively. The darker region on the cantilever beam corresponds to the metal–piezo–metal stack covered region. A reflecting Au disk in the center was designed for additional laser beam deflection tests. As shown in both SEM images, the diameter of the seismic mass is decreasing from the back side towards the membrane, i.e., the sidewall of the DRIE etching was not perpendicular. By a closer SEM observation, the cone angle was found to be 7°.

**Figure 5:**
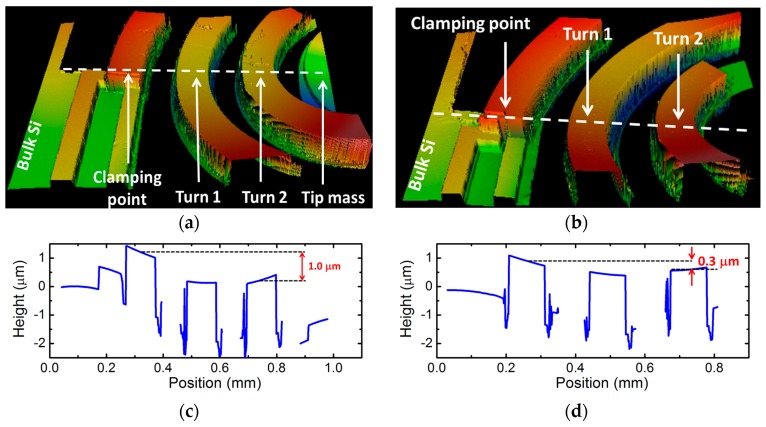
3D optical surface profiler images taken on a portion of a suspended spiral with (**a**) and without (**b**) seismic mass at its end. Characteristic height profiles along the dashed lines for cantilever with (**c**) and without seismic mass (**d**).

AlN thin films often have a significant compressive or tensile residual stress which depends on several factors, such as the deposition technique (RF sputtering, pulsed DC sputtering, chemical vapor deposition (CVD), etc.), growth parameters (flow rate, deposition temperature, plasma power, etc.) [27], the material quality of the underlaying template [28], or the layer thickness [29]. In our previous studies, we found that AlN layers having a similar thickness deposited directly on Si wafer in our RF sputtering system resulted in a significant tensile stress (~575 MPa) in the layer. In contrast, the freely suspended spiral cantilevers seem to be flat in the SEM images (Figure 4); i.e., neither significant downward deflection due to gravity, nor upward deformation due to tensile stress, is visible. Nevertheless, in order to quantify the strain, we performed tests of optical surface profile on two cantilevers. The first one was an intact spiral of an array position of (1,1) with tip mass. From the second chip (4,3), the tip mass was intentionally removed to study the effect of the residual stress directly. Figure 5a,b show the corresponding 3D images recorded on one side of the spirals by a 10× objective lens. Height line profiles taken along the dashed lines for the cantilever, with and without tip mass, are shown in Figure 5c,d, respectively. As shown in Figure 5a, though the tip mass is tilted due to its torsional moment, the height difference between the clamping point and in the same *y* position in the second turn is 1.0 μm, though the arc length between the two points is about 6 mm. Without tip mass, the deflection is even smaller, and the height changes around 0.3 μm along an arc length of about 9.3 mm. This indicates that the stress in AlN on the spiral is negligible, and the deflection is mainly affected by the static load of the central seismic mass. This low residual stress, compared to our previously found results, can be attributed either to the beneficial effect of the Au bottom contact upon sputter deposition, or to the device geometry.

### 3.2. Vibration Tests and Validation of the Analytical Model

**Figure 6:**
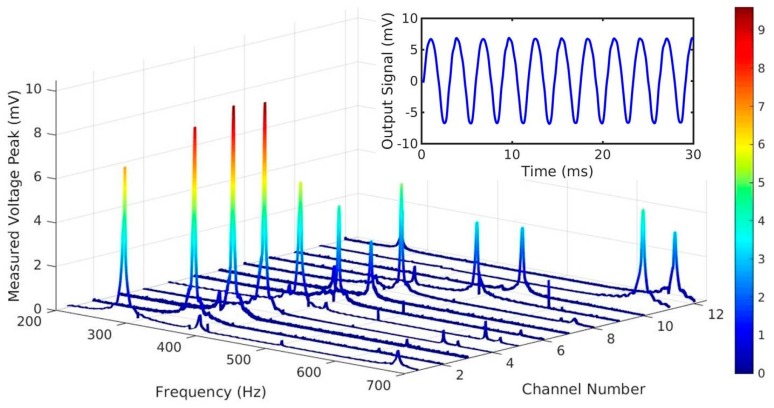
Piezoelectric output voltage during continuous sinusoidal excitation at a feedback controlled acceleration of 1 g. Depending on the geometry, the base frequency of the cantilevers falls in the range of 281–673 Hz. Inset shows the sinusoidal output waveform of channel 1 at resonance.

Vibration tests, performed at a fixed acceleration of 1 g (9.81 m/s^2^), confirmed the frequency selectivity of the cantilever array (Figure 6). Most of the spirals (12 out 16) showed sharp resonance peaks scattering in the range of 281–672 Hz. For clarity, the spectra were ordered by their resonance frequencies from low to high (Channel 1–12). The calculated Q-factors $\left(f_{0}/\Delta f_{\sqrt{2}}\right)$ in air vary in the range of 117–254. As shown in the inset image of Figure 5, the recorded time-dependent open circuit signal is purely sinusoidal, without any vertical offset, which indicates stress-free cantilevers. The inactivity of the remaining four devices can be attributed to the low electrical resistance of the metal/AlN/metal stacks, which may be the results of random defects in the AlN layer. The generated open circuit voltages fell in the range of 3.0–9.6 mV.

Using the analytical method described in Section 2.2, resonance frequencies were calculated for each cantilever by taking into account the tapered geometry of the seismic mass, and the layer stack on the top of the cantilever. The obtained frequency values have good agreement with the experimental ones; the calculated root-mean-square deviation is 21.5 Hz. Table 1 summarizes the geometrical parameters, the experimentally and numerically obtained resonance frequencies, as well as the generated V_OC_ amplitude for each cantilever.

In order to validate the analytical model, and to see if any residual stress in the coating causes systematic shift in the resonance frequency, we performed vibration tests on coating free bare cantilevers. For that we removed the SiO_2_/Ti/Au/AlN/Ti/Au stacks from one chip in subsequent etching steps of potassium iodide solution, hot phosphoric acid (85 °C), potassium iodide solution, and hydrofluoric acid. The resonance frequencies of the bare non-piezoelectric cantilevers were determined by eye under optical microscope at manual frequency sweeps. As expected, the obtained natural resonance frequencies are lower than the corresponding values for coated cantilevers. However, it is a question if this 14.5% average lowering in frequency can be ascribed solely to the softening of the cantilever or whether stress relaxation is also considerable. Therefore, we removed the layer stack from the model as well, and repeated the calculation with the average Si Young’s modulus value of *E_Si_* = 149.5 GPa. As shown in the last two columns of Table 1, the agreement between the measured and calculated resonance frequencies for bare Si cantilevers is excellent. The obtained root-mean-square deviation of 5.8 Hz is remarkably low for such a simplified analytical model. It has to be noted that no fitting parameter was used for the calculation, only the measured geometrical and literature material parameters were used. Since the agreement was also good for the coated cantilevers, and no systematic shift was observed in the resonance frequencies, we can conclude that the effect of the AlN stress on the resonance frequency, if there is any, is negligible in these cantilevers.

### 3.3. Resonant Modes

**Figure 7:**
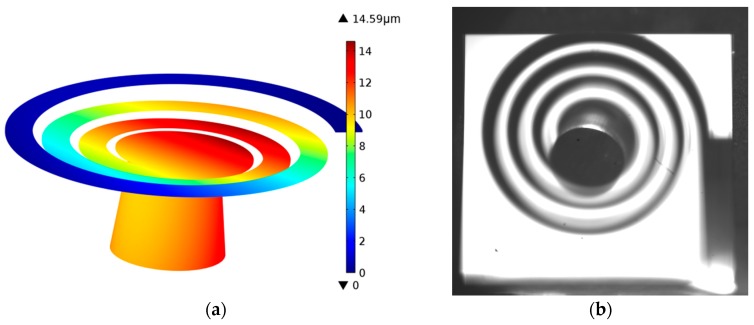
(**a**) Deflection distribution along the spiral cantilever at a driving acceleration of 1 g calculated by finite element analysis; (**b**) stroboscopic snapshot taken from the backside during shaking of the microspiral under optical microscope. The live slow-motion video clearly showed the off-axis wobbling movement of the seismic mass.

As it was found by the detailed COMSOL analysis, the listed eigenfrequencies do not correspond to a pure vertical oscillation of the seismic mass (bending mode) but are also accompanied with a torsional vibration. As shown in Figure 7a, the displacement of the beam is not simply increasing from the clamping point to the center, but also superimposed with a periodic fluctuation. The off-axis wobbling motion of the mass was also confirmed by a stroboscopic observation of the resonating cantilever under optical microscope (Figure 7b).

**Figure 8:**
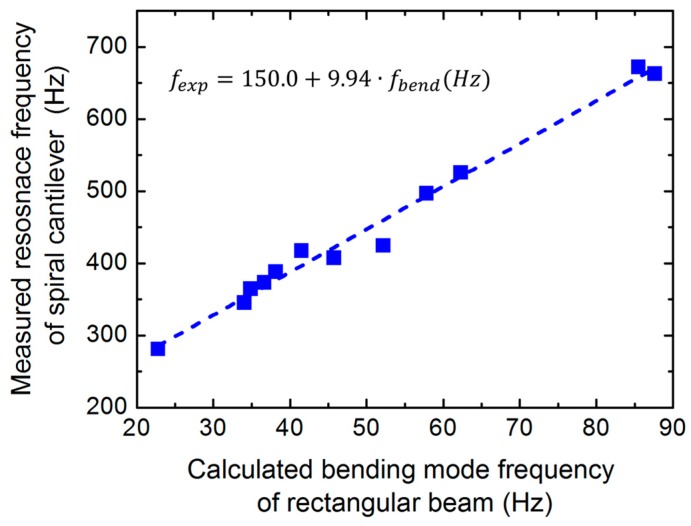
Experimental resonance frequencies for each cantilever as a function of the calculated first bending mode frequency using a simplified rectangular beam model.

In accordance with these findings, the first bending mode frequencies calculated by a simplified straight rectangular model (Equation (2)) using the same length, width (Table 1), and thickness parameters are significantly lower than the corresponding experimental values shown in Figure 6. Nevertheless, by plotting these points against each other, a clear linear correlation is observed (*f*_exp_ = 150 + 5.94∙*f*_bend_), which can be used as a simple guideline for further chip designs (Figure 8). Repeated vibrational analyses performed in the range of 20–200 Hz revealed the presence of lower frequency resonances, presumably corresponding to these bending modes. However, they produce significantly lower voltage signals, which are hardly detectable in the background electrical noise.

### 3.4. Applicability in FICI

In the following, we will discuss the applicability of the piezo MEMS spirals in next generation FICI from the viewpoint of the tonotopy and the output voltage. The test arrays showed clear frequency selectivity in the 281–672 Hz range, which corresponds to the lowest part of the voice frequency regime. The high Q-factors, up to 254, enable high vibration sensitivity and frequency selectivity. By applying a shorter cantilever, it can be easily extended to the whole range up to 3400 Hz, or even to 20 kHz at the same or at a smaller device footprint. The ultralow frequency range down to the hearing limit of 20 Hz is more challenging, but seems to be also feasible for a 2 mm × 2 mm cantilever, assuming that we can enhance the torsion free vertical oscillating mode e.g., by a double beam sandwich structure [12]. One critical issue, which can cause false tones for a CI patient, is an unwanted overtone. With the demonstrated design, its exclusion was fairly successful in the 20–1200 Hz frequency range, since only very small second peaks are visible in Figure 6, and the ultralow frequency resonances were suppressed. However, the cantilever response in the upper part of the human audible range (1.2–20 kHz) was not investigated in the present study.

The obtained output voltage signals (3–10 mV) are higher than the ones reported by Jang et al. [11], therefore, it must be sufficient to excite hearing nerves through an implantable amplifier using a similar system. However, in this study, the excitation was not done directly by sound pressure, but by controlled acceleration of the cantilever frame. Moreover, its magnitude is much higher than the typical values for human ossicles. The displacement of the stapes footplate is about 30 nm at 500 Hz, and at a sound pressure level of 90 dB stimulus [17], which corresponds to an acceleration of 0.30 m/s^2^. Assuming a linear relationship between the output voltage and the acceleration, the corresponding Channel 9 oscillator would result in an open circuit voltage of 100 μV, which is comparable to the magnitude which was detected and amplified by Jang et al. [11]. On the other hand, the ultimate solution of a FICI is a self-powered artificial basilar membrane which generates sufficient electronic power for the direct excitement of the hearing nerves through an implanted multielectrode. However, it requires further optimization in biocompatible piezoelectric materials, in CI electrode stimulation efficiency, and in implantation design.

## 4. Conclusions

In summary, we demonstrated a one arm spiral cantilever design and fully CMOS compatible fabrication procedure using biocompatible AlN piezoelectric layer and 3D micromachining. As it was confirmed by surface optical microscope and vibration tests, the AlN coated cantilevers are free of stress, which is essential for highly demanding human implants. The fabricated piezoelectric spiral array with Si tip mass exhibited a clear tonotopy in the lower audible frequency range (281–672 Hz). The obtained high Q-factors (117–254) ensure high frequency selectivity for the targeted hearing sensor. To the analytical model of Karami et al. [23], we added extra terms to represent the seismic mass at the end of the spiral shape beam. The calculated natural resonance frequencies showed excellent agreement with the experimental values for coating free cantilevers (RMS = 5.8 Hz). The same method was also successfully used on AlN coated cantilevers, using a composite Young’s modulus value for the layer stack. Besides, we also obtained a simple fit equation which helps to predict the main resonance frequencies of the spiral beams. All the manufactured cantilevers fit into a square of 2 mm × 2 mm, which satisfies the size constraint for middle ear implants. Thus, the feasibility of a frequency selective compact sensor array has been demonstrated that could provide a basis for further fully implantable cochlear implants.

## Figures and Tables

**Table 1:** Geometrical parameters, calculated and measured resonance frequencies, peak output voltages, quality factors for each piezoelectric cantilever, as well as the resonance frequency of uncoated spiral beam and the corresponding theoretical values.

Ch.	L	w	R_0_	r	m_beam_	m_tm_	Res. Freq.		Peak	Q	Res. Fr./Etched	
No.							Exp.	Calc.	Voltage	Factor	Exp.	Calc.
	(π/mm)	(μm)	(μm)	(μm)	(ng)	(ng)	(Hz)	(Hz)	(mV)		(Hz)	(Hz)
1	8.0/11.8	145	870	315	50	320	281	283	6.89	145	241	240
2	7.0/9.4	150	850	300	41	287	345	337	8.80	133	289	295
3	7.0/9.2	150	800	300	40	287	365	366	9.60	148	311	312
4	7.0/9.2	150	800	290	40	266	374	348	9.53	254	321	319
5	7.0/9.1	145	800	280	38	246	389	377	5.71	117	335	332
6	6.5/7.4	160	760	310	36	309	408	379	4.47	218	343	348
7	8.0/10.3	145	800	250	40	190	417	424	2.64	176	352	357
8	6.0/7.4	160	760	300	33	287	425	473	5.02	216	- ^2^	362
9	7.0/7.7	160	760	250	36	190	497	471	3.40	180	422	424
10	7.0/7.7	150	760	230	34	157	526	543	3.05	173	449	449
11	6.5/6.3	180	700	230	34	157	663	679	4.40	245	578	576
12	6.0/6.5	140	700	210	27	127	672	687	3.14	210	590	574
13	7.0/9.9	145	850	310	42	309	^1^	363	-	-	268	-
14	8.0/9.6	140	760	230	40	157	^1^	482	-	-	414	-
15	6.0/6.6	180	700	180	27	88	^1^	874	-	-	707	-
16	6.0/6.4	140	700	160	26	66	^1^	1058	-	-	^2^	-

^1^ No resonance frequency was found in the 200–1200 Hz range. ^2^ Cantilever was broken during etching.

## References

[1] Wilson B.S., Dorman M.F. (2008). Cochlear implants: Current designs and future possibilities. J. Rehabil. Res. Dev..

[2] Cohen N. (2007). The totally implantable cochlear implant. Ear Hear..

[3] Zhou G., Bintz L., Anderson D.Z., Bright K.E. (1993). A life-sized physical model of the human cochlea with optical holographic readout. J. Acoust. Soc. Am..

[4] Wittbrodt M.J., Steele C.R., Puria S. (2006). Developing a physical model of the human cochlea using microfabrication methods. Audiol. Neurotol..

[5] White R.D., Grosh K. (2005). Microengineered hydromechanical cochlear model. Proc. Natl. Acad. Sci. USA.

[6] Shintaku H., Nakagawa T., Kitagawa D., Tanujaya H., Kawano S., Ito J. (2010). Development of piezoelectric acoustic sensor with frequency selectivity for artificial cochlea. Sens. Actuators Phys..

[7] Inaoka T., Shintaku H., Nakagawa T., Kawano S., Ogita H., Sakamoto T., Hamanishi S., Wada H., Ito J. (2011). Piezoelectric materials mimic the function of the cochlear sensory epithelium. Proc. Natl. Acad. Sci. USA.

[8] Harada M., Ikeuchi N., Fukui S., Ando S. (1998). Fish-bone-structured acoustic sensor toward silicon cochlear systems. Micromachined Devices and Components IV.

[9] Xu T., Bachman M., Zeng F.-G., Li G.-P. (2004). Polymeric micro-cantilever array for auditory front-end processing. Sens. Actuators Phys..

[10] Jang J., Kim S., Sly D.J., O’leary S.J., Choi H. (2013). MEMS piezoelectric artificial basilar membrane with passive frequency selectivity for short pulse width signal modulation. Sens. Actuators Phys..

[11] Jang J., Lee J., Woo S., Sly D.J., Campbell L.J., Cho J.-H., O’Leary S.J., Park M.-H., Han S., Choi J.-W. (2015). A microelectromechanical system artificial basilar membrane based on a piezoelectric cantilever array and its characterization using an animal model. Sci. Rep..

[12] Choi W.J., Jeon Y., Jeong J.-H., Sood R., Kim S.G. (2006). Energy harvesting MEMS device based on thin film piezoelectric cantilevers. J. Electroceram..

[13] Zhang L., Lu J., Takei R., Makimoto N., Itoh T., Kobayashi T. (2016). S-shape spring sensor: Sensing specific low-frequency vibration by energy harvesting. Rev. Sci. Instrum..

[14] Lu J., Zhang L., Yamashita T., Takei R., Makimoto N., Kobayashi T. (2015). A silicon disk with sandwiched piezoelectric springs for ultra-low frequency energy harvesting. J. Phys. Conf. Ser..

[15] Jackson N., Keeney L., Mathewson A. (2013). Flexible-CMOS and biocompatible piezoelectric AlN material for MEMS applications. Smart Mater. Struct..

[16] Heidrich N., Knöbber F., Sah R.E., Pletschen W., Hampl S., Cimalla V., Lebedev V. Biocompatible AlN-based piezo energy harvesters for implants. Proceedings of the 2011 16th International Solid-State Sensors, Actuators and Microsystems Conference.

[17] Gan R.Z., Dai C., Wang X., Nakmali D., Wood M.W. (2010). A totally implantable hearing system—Design and function characterization in 3D computational model and temporal bones. Hear. Res..

[18] Beker L., Zorlu Ö., Göksu N., Külah H. Stimulating auditory nerve with MEMS harvesters for fully implantable and self-powered cochlear implants. Proceedings of the 2013 Transducers & Eurosensors XXVII: The 17th International Conference on Solid-State Sensors, Actuators and Microsystems (TRANSDUCERS & EUROSENSORS XXVII).

[19] Ansari M.Z., Cho C. (2009). Deflection, Frequency, and Stress Characteristics of Rectangular, Triangular, and Step Profile Microcantilevers for Biosensors. Sensors.

[20] Sharpe W.N., Yuan B., Vaidyanathan R., Edwards R.L. Measurements of Young’s modulus, Poisson’s ratio, and tensile strength of polysilicon. Proceedings of the IEEE Tenth Annual International Workshop on Micro Electro Mechanical Systems. An Investigation of Micro Structures, Sensors, Actuators, Machines and Robots.

[21] Stokey W.F. (2002). Vibration of systems having distributed mass and elasticity. Harris’ Shock and Vibration Handbook.

[22] Sooriakumar K., Chan W., Savage T.S., Fugate C. (1995). A Comparative Study of Wet vs. Dry Isotropic Etch to Strengthen Silicon Micromachined Pressure Sensor. A Comparative Study of Wet vs. Dry Isotropic Etch to Strengthen Silicon Micromachined Pressure Sensor.

[23] Karami M.A., Yardimoglu B., Inman D. Coupled Out of Plane Vibrations of Spiral Beams. Proceedings of the 50th AIAA/ASME/ASCE/AHS/ASC Structures, Structural Dynamics, and Materials Conference.

[24] Shackelford J.F., Alexander W. (2000). Mechanical Properties of Materials. CRC Materials Science and Engineering Handbook.

[25] Timoshenko S., Young D.H. (1962). Elements of Strength of Materials.

[26] Hopcroft M.A., Nix W.D., Kenny T.W. (2010). What is the Young’s Modulus of Silicon?. J. Microelectromech. Syst..

[27] Zhong H., Xiao Z., Jiao X., Yang J., Wang H., Zhang R., Shi Y. (2012). Residual stress of AlN films RF sputter deposited on Si(111) substrate. J. Mater. Sci. Mater. Electron..

[28] Dubois M.-A., Muralt P. (2001). Stress and piezoelectric properties of aluminum nitride thin films deposited onto metal electrodes by pulsed direct current reactive sputtering. J. Appl. Phys..

[29] Pobedinskas P., Bolsée J.-C., Dexters W., Ruttens B., Mortet V., D’Haen J., Manca J.V., Haenen K. (2012). Thickness dependent residual stress in sputtered AlN thin films. Thin Solid Films.

